# Education in Laparoscopic Cholecystectomy: Design and Feasibility Study of the LapBot Safe Chole Mobile Game

**DOI:** 10.2196/52878

**Published:** 2024-07-25

**Authors:** Mohammad Noroozi, Ace St John, Caterina Masino, Simon Laplante, Jaryd Hunter, Michael Brudno, Amin Madani, Marta Kersten-Oertel

**Affiliations:** 1 Applied Perception Lab Department of Computer Science and Software Engineering Concordia University Montreal, QC Canada; 2 University of Maryland Medical Center Baltimore, MD United States; 3 Surgical Artificial Intelligence Research Academy University Health Network Toronto, ON Canada; 4 Department of Surgery University of Toronto Toronto, ON Canada; 5 DATA Team University Health Network Toronto, ON Canada

**Keywords:** gamification, serious games, surgery, education, laparoscopic cholecystectomy, artificial intelligence, AI, laparoscope, gallbladder, cholecystectomy, mobile game, gamify, educational game, interactive, decision-making, mobile phone

## Abstract

**Background:**

Major bile duct injuries during laparoscopic cholecystectomy (LC), often stemming from errors in surgical judgment and visual misperception of critical anatomy, significantly impact morbidity, mortality, disability, and health care costs.

**Objective:**

To enhance safe LC learning, we developed an educational mobile game, LapBot Safe Chole, which uses an artificial intelligence (AI) model to provide real-time coaching and feedback, improving intraoperative decision-making.

**Methods:**

LapBot Safe Chole offers a free, accessible simulated learning experience with real-time AI feedback. Players engage with intraoperative LC scenarios (short video clips) and identify ideal dissection zones. After the response, users receive an accuracy score from a validated AI algorithm. The game consists of 5 levels of increasing difficulty based on the Parkland grading scale for cholecystitis.

**Results:**

Beta testing (n=29) showed score improvements with each round, with attendings and senior trainees achieving top scores faster than junior residents. Learning curves and progression distinguished candidates, with a significant association between user level and scores (*P*=.003). Players found LapBot enjoyable and educational.

**Conclusions:**

LapBot Safe Chole effectively integrates safe LC principles into a fun, accessible, and educational game using AI-generated feedback. Initial beta testing supports the validity of the assessment scores and suggests high adoption and engagement potential among surgical trainees.

## Introduction

Laparoscopic cholecystectomy (LC; ie, gallbladder removal) is one of the most commonly performed procedures in the United States and across the world [1]. A complication of LC is bile duct injuries, which can lead to significant morbidity, mortality, disability, and, in turn, health care costs [2-5]. These injuries are often due to errors in surgical judgment and visual misperceptions of critical anatomy and tissue planes [5]. The goal of this work is to develop a game that may improve trainees’ perception and mental models of dissection planes and anatomy in the context of LC to enhance their performance during LC and help avoid injuries. Specifically, we have developed a game, “LapBot Safe Chole” to study the impact of feedback from artificial intelligence (AI) models on learning safe and dangerous zones of dissection (Go or No-Go) in LC.

In the surgical domain, AI models can be trained to perform computer vision tasks (eg, identify the anatomical structure and interpret scenes in the surgical field) and therefore potentially provide guidance, navigational data, and decision support to surgeons to reduce errors that lead to complications. For example, Madani et al [1] developed deep learning models to identify Go and No-Go zones of dissection, liver, gallbladder, and hepatocystic triangle during LC. In their work, deep learning was used to develop an AI model to accurately identify Go and No-Go zones. The predicted Go and No-Go zones can then be visualized on surgical scenes using augmented reality (AR) for training or surgical guidance. With AR virtual elements are overlaid onto the real world. In our case, the virtual element is the visual representation of the AI model results of where it is safe and unsafe to dissect (ie, Go and No-Go zones), and these are overlaid with AR onto the real surgical scene. These annotated videos are used as feedback in our game app to determine the impact of feedback on learning curves.

Games have proven to be effective educational tools in numerous domains and across varying skill levels. Game-based learning can promote student engagement, allow for risk-taking, and promote collaborative learning [6]. In surgical education, serious games have been shown to improve intraoperative skills and decision-making and can be formally incorporated into the official curriculum for individual residency programs [7]. Using games in surgical education is essential for promoting deliberate practice [4], that is, repeatedly performing a specific activity in low-pressure conditions to better use the skill in high-stakes situations, such as surgical decision-making in the operating room. Such practice is critical to achieving expert proficiency, particularly in performance-based disciplines [8]. A critical element of deliberate practice is expert feedback and coaching. Modern technological advancements, including the introduction of AI for coaching, have potentially made gaming even more beneficial. In this way, if an algorithm can be trained to replicate the mental model of expert surgeons, then it can be leveraged to provide feedback anywhere, at any time, and reach many individuals simultaneously. Ultimately, this would overcome one of the greatest obstacles to surgical coaching, which is the availability of faculty for consistent and timely feedback.

The field of educational game apps has witnessed significant advancements in recent years, with various studies exploring the effectiveness of innovative tools for training and assessment purposes. The integration of serious gaming in health care, such as virtual reality (VR) training, has also shown promising results in increasing intrinsic motivation. For example, Touch Surgery (Medtronic) is a mobile platform that offers training and self-assessment modules for various procedures, which are incorporated into a game-like structure. The app provides step-by-step guidance and assesses cognitive decision-making through multiple-choice questions. A study aimed to establish the validity of the LC module within Touch Surgery showed the importance of multimedia learning tools and proficiency-based training in LC surgical education [9]. Additionally, a pilot trial investigated the potential synergy between Touch Surgery and VR trainers in reducing learning curves. The findings showed the need for automated feedback and evaluation systems to enhance training efficiency and advocated for a multimodal training approach that combines Touch Surgery with VR trainers to optimize laparoscopy training [9]. Overall, the study concluded that the cost-free Touch Surgery app is an innovative tool for training and assessment. Several other studies have looked at using games to learn laparoscopic skills including the works of IJgosse et al [10,11] and Jalink et al [12], who used a Wii game for a warm-up for surgery and a Nintendo game for looking a performance [13]. Other serious games in surgical and clinical contexts include a game for teaching chest tube insertions [14], setting up surgical instruments [15], and learning uterine artery ligation surgery [16]. The interested reader can read more about the use of serious games for improving skills in the clinical domain in a scoping review by Olgers et al [17].

The effectiveness of learning through mobile apps depends on several factors. These include engagement, cognitive activity, goal-oriented guidance, and the incorporation of scaffolding and interactive learning activities [18]. For older users, such as our user base, there are additional factors that may impact learning patterns and behaviors, including motivation, expectation, and prior experiences [19]. It is important to consider all these elements when designing mobile apps to enhance the overall experience and retention of learners or trainees. Numerous studies have shown that immediate feedback as well as thoughtfully designed game elements can positively impact motivation, enjoyment, and learning [20-22].

The purpose of the research presented in this paper was to assess the feasibility of developing an AI-generated feedback game to facilitate learning of safe LC. The specific focus of this paper is to describe the motivation of the game as well as the development of the game that was aimed to be engaging, interactive, and motivating. To do so, we carefully designed gameplay elements, such as levels and scoring, as well as immediate feedback. Furthermore, our study provides the LapBot game with its first evaluation.

## Methods

In the following section, we describe the development and features of the game followed by the user study description.

### LapBot Safe Chole

The LapBot Safe Chole app was developed using React Native, an open-source user interface software framework created by Meta Platforms. Both the front-end and backend sides of LapBot were developed with React, and the server side was developed using PHP. All the requests from the app to the server are sent by the Fetch API (a recent version of XMLHttpRequest).

**Figure 1:**
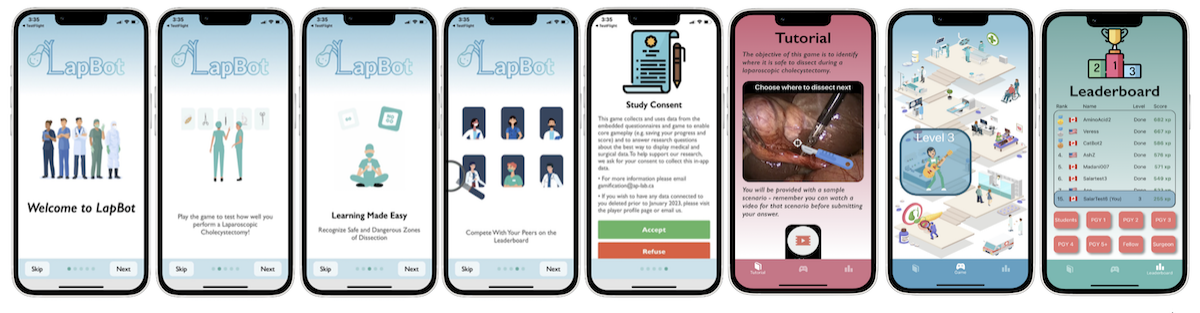
Screenshots of the LapBot game. Left to right: Onboarding, study consent, tutorial, level screen, and leaderboard of the LapBot game.

After downloading the game, at the first launch of the app, users will see the onboarding screens of LapBot (Figure 1). The onboarding screens introduce users to the app and describe the main goals of LapBot. Prior to gameplay, the users are required to create an account, consent to data collection, and fill out a short demographic survey. Participants are asked to indicate whether they are medical students, postgraduate students in years 1 to 4, surgical fellows, or attending surgeons. This information is reflected in the leaderboard for the game. After creating the account, there are 3 main dashboard screens: the tutorial, level board, and leaderboards. The tutorial is provided to the user with gameplay instructions prior to starting level 1.

### Gameplay Elements

LapBot has 5 levels, where the level of difficulty corresponds to the Parkland grading scale for cholecystitis [23]. The Parkland grading of each of the surgical scenarios was done by 2 surgeons who divided the rounds into 5 different levels of the game. Each level contains 20 rounds; however, to pass a level, the player needs to pass 5 consecutive rounds with a minimum threshold score of greater than 50%. There are 5 gallbladder icons in the top right of the screen indicating to the player how many consecutive rounds they have passed in the current level. If at any point a player fails an individual round, the gallbladder counter resets to 0, and the player must restart that level.

**Figure 2:**
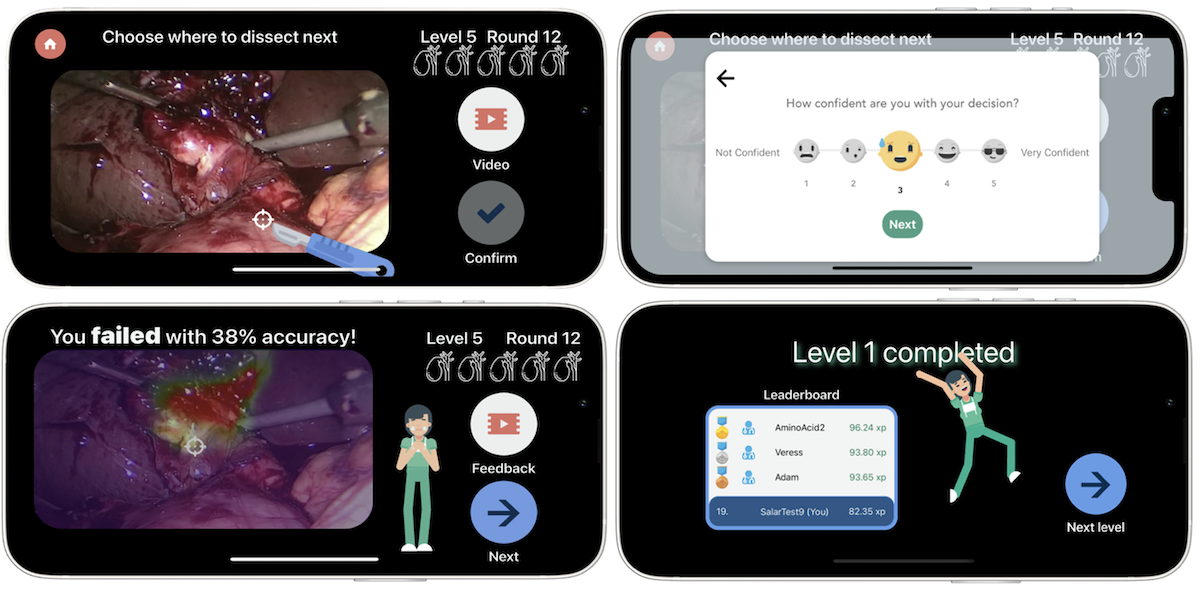
Gameplay screens. Top left: The player chooses where the optimal location of dissection is based on the video frame. Players can play a video of 5 seconds prior to this video frame by clicking “Video.” Once they choose, a pop-up asking them about their level of confidence in their decision is shown (top right). Next, they see their results and the artificial intelligence–annotated Go zone (bottom left). They need to pass 5 consecutive rounds (gallbladders shown in the top right) to finish a level (bottom right).

**Figure 3:**
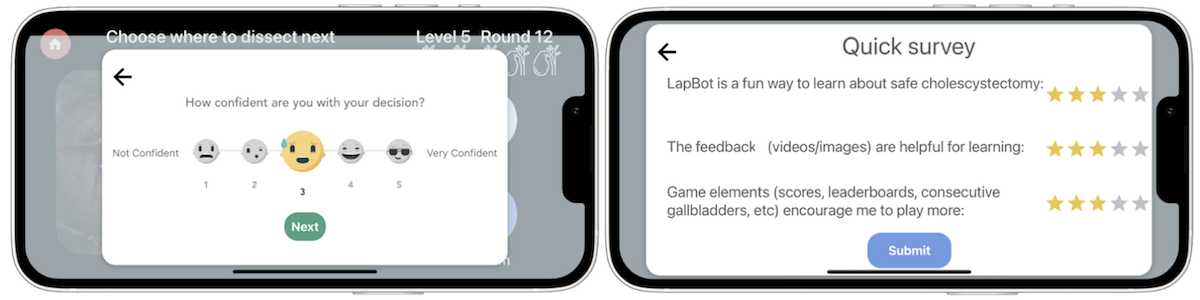
Screenshots of 2 quick in-app surveys: the left screen appears after each answer that the player submits to determine their level of confidence with each chosen target. The right survey screen only appears once after finishing level 2 and asks the users about their experience using LapBot.

In each round of the game, the player is presented with a surgical frame or video from a real surgery, and their task is to choose where they would dissect next with consideration of a safe dissection zone (Figure 2). To understand the surgical context of the frame they are presented, the player can watch up to 5 seconds of the surgical video prior to the given frame by clicking on the “Video” button. After the target is confirmed by the user (by clicking the “Confirm” button), the player is presented with a pop-up asking how confident they are with their answer. Next, they are presented with feedback that depicts their chosen target point with respect to the AI annotations of safe (Go) and dangerous (No-Go) zones of dissection given the surgical frame (Figure 3). The player can also choose to watch the video with the AI annotation overlaid in an AR view on all the frames to get additional feedback by clicking on the “Feedback” button.

**Figure 4:**
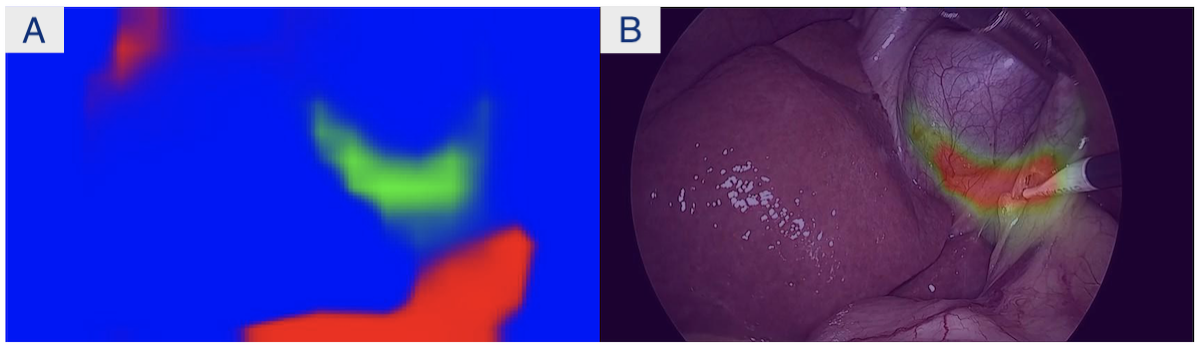
(A) The artificial intelligence (AI) output of the frame that is used to calculate the user’s score. The 3 colors, green, red, and blue, denote the Go zones, No-Go zones, and neutral zones, respectively. The right frame provides a visualization of the Go zone (ie, the ideal answer to where they should have dissected in the given scenario). (B) Augmented reality heatmap showing the Go zone. Note that the Go zone is depicted as a heatmap (from red through orange, yellow, and green); with red denoting high confidence of the AI model that this is a safe zone to dissect, and green lower confidence that this is a safe zone.

Leaderboards have been shown to be effective in improving learning motivation and promoting learning in gamified contexts. Thus, based on the results of Park and Kim [24], we provide users with both a macro (overall leaderboard) as well as a micro (associated with each level) as seen in Figure 1 last screenshot and Figure 2 bottom right image, respectively.

### Study Design

We performed a retrospective study based on beta testing data over a period from November 2022 to December 2022. The goal of the beta testing was to evaluate the usability and effectiveness of the game design elements in terms of surgical education in LC. A convenience sample was used for the recruitment of medical students, resident trainees, fellows, and surgeon consultants.

### Ethical Considerations

The mobile app was offered free of charge. The study was approved by the Concordia University Research Ethics Board (30016074). Informed consent was requested after onboarding of the game. Gameplay data were linked to the player’s demographic data; however, participants always had the option to opt out of the study (information on how to do this was provided in the app itself). No compensation was provided.

### Statistical Analysis

Data management and statistical analyses were performed using SAS software (version 9.4; SAS Institute). For qualitative variables, responses were grouped into congruent themes, and the corresponding response rates were computed and expressed as percentages. Quantitative variables were carefully examined through univariate analysis, and the outcomes were likewise reported in percentage terms. Any data incompleteness was thoroughly evaluated, with the extent of missing answers considered negligible and thus not impacting the conclusions. For statistical comparisons, the Mann-Whitney *U* test was applied to categorical variables, while ANOVA was used to compare continuous variables between more than 2 groups. Furthermore, multivariate linear regression was used to estimate the adjusted relationships between the end score, postgraduate year (PGY), and participants’ self-perceived confidence in their abilities. Throughout our analysis, a *P*<.05 was adhered to as the threshold for statistical significance.

## Results

### Participants

In the following section, we describe the results related to the effectiveness of the design and features of the game. A total of 29 participants participated in the usability testing phase, of which, 14 (48%) completed all 5 levels of the game. Of the 14 participants who completed the game, 2 were medical students, 2 were PGY1 trainees, 4 were PGY3 trainees, 1 was a PGY4 trainee, 2 were clinical fellow surgeons, and there were 3 attending surgeons. This diverse cohort of participants provided insights into the usability and potential of the game, facilitating an assessment of its effectiveness. When looking into when participants quit, we found that 4 participants quit after the tutorial, 3 after failing to complete a round, and 8 after successfully completing a level.

### Game Level Evaluation

**Figure 5:**
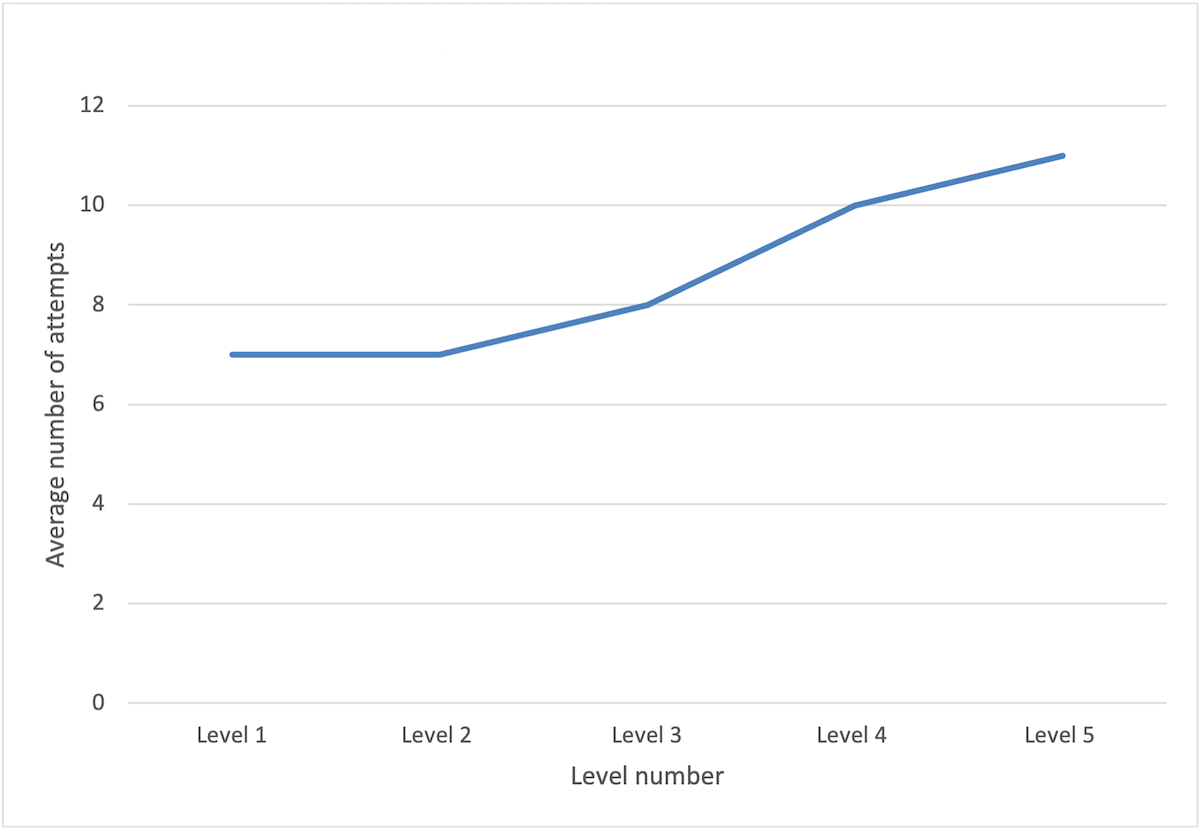
An analysis of player performance in terms of rounds needed to pass a level across varying difficulty levels in the LapBot game.

The intention behind the game design was to increase the difficulty of the game as users progressed through the 5 levels. To accomplish this, the ordering of the levels within the game was carried out by expert surgeons to ensure that the content progression was tailored to reflect the real-world expertise and skill development of surgeons. As can be seen in Figure 5, players indeed encountered greater difficulties in passing the higher levels, aligning with the intended design. The graph shows the average number of attempts users need to successfully pass each level, with a clear upward trend from level 1 to level 5. This outcome indicates a successful implementation of the game’s progression structure, achieving the desired goal of presenting users with increasing challenges as they advance.

### Player Confidence

**Figure 6:**
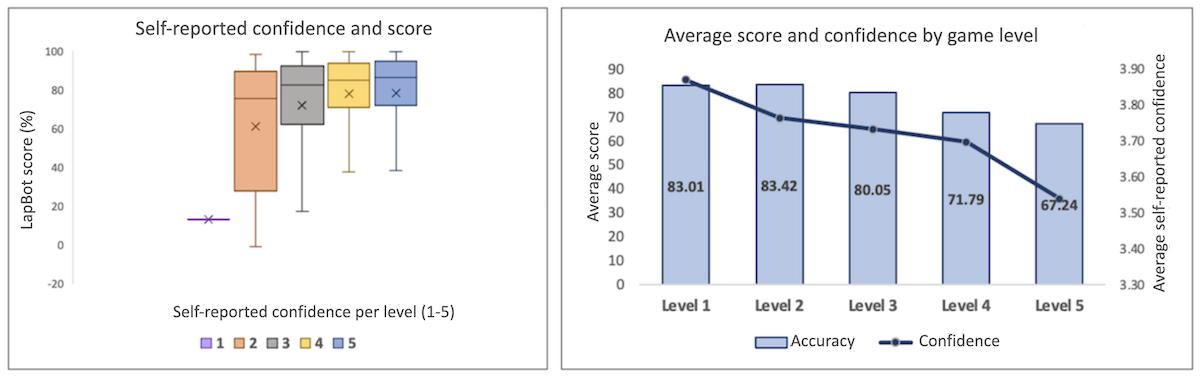
Left: Users who expressed higher levels of confidence in their answers achieved better scores. Right: As the level increased, the average confidence decreased.

In terms of player confidence, captured through the pop-up question after each round, as expected, we found a positive and significant correlation between self-assessed confidence level and score (*P*<.001). Users who expressed higher levels of confidence in their answers achieved better scores, while those who displayed lower confidence obtained comparatively lower scores (Figure 6, left). In other words, users were able to adequately self-assess predicted performance. We also found that as the level increased, there was a stepwise decline in the average score, accompanied by a corresponding decrease in participants’ average confidence (Figure 6, right panel).

### Performance

**Figure 7:**
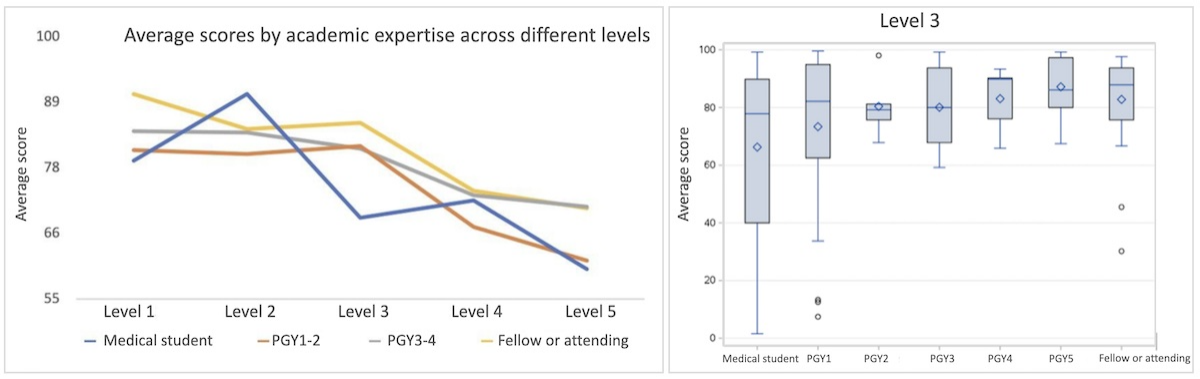
Left: Distinct lines representing different academic expertise grades across the 5 levels. Right: Scores for level 3, a clear stepwise pattern is observed, with more experienced users achieving higher scores. PGY: postgraduate year.

We found a positive and statistically significant association between the level of user and their scores (*P*=.003). Specifically, more experienced players tend to achieve higher scores on average. In looking at the relationship between experience and corresponding scores, we found a consistent pattern where users with higher levels of academic expertise consistently outperformed those with less experience. As an example, we show level 3 performance in Figure 7. Notably, the scores obtained by medical students are lower compared to those of PGY1 residents, and this trend continues with increasing academic expertise with attending surgeons achieving the highest scores.

**Figure 8:**
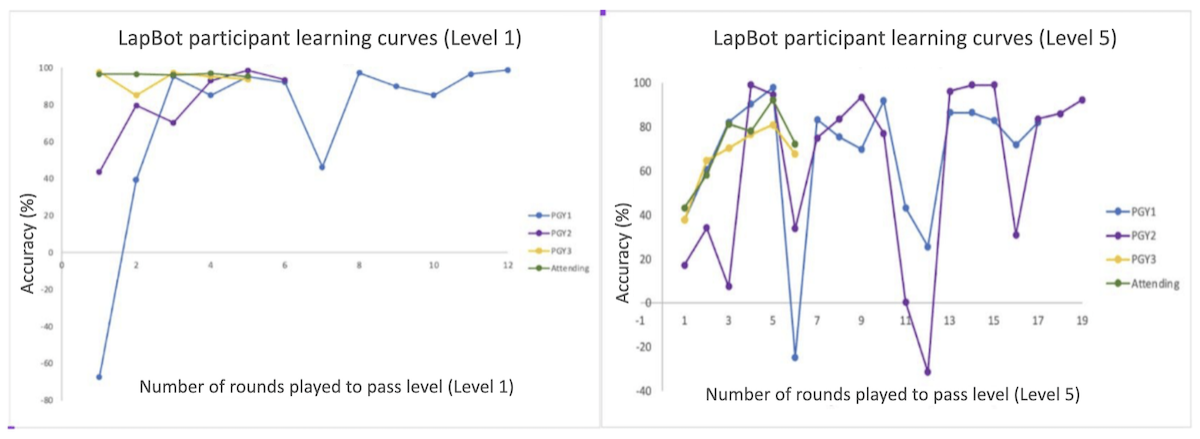
Player performance across (left) level 1 and (right) level 5. PGY: postgraduate year.

We also looked at the players’ average scores as they progressed through the game’s levels. This progression is particularly evident when analyzing the data for each level separately. We found that players with lower academic expertise begin with lower scores but exhibit a significant improvement in their performance as they continue playing and learning from the game. On the other hand, more experienced players, such as attending surgeons, start with higher scores from the outset and maintain a consistently high level of performance throughout the level (Figure 8).

**Figure 9:**
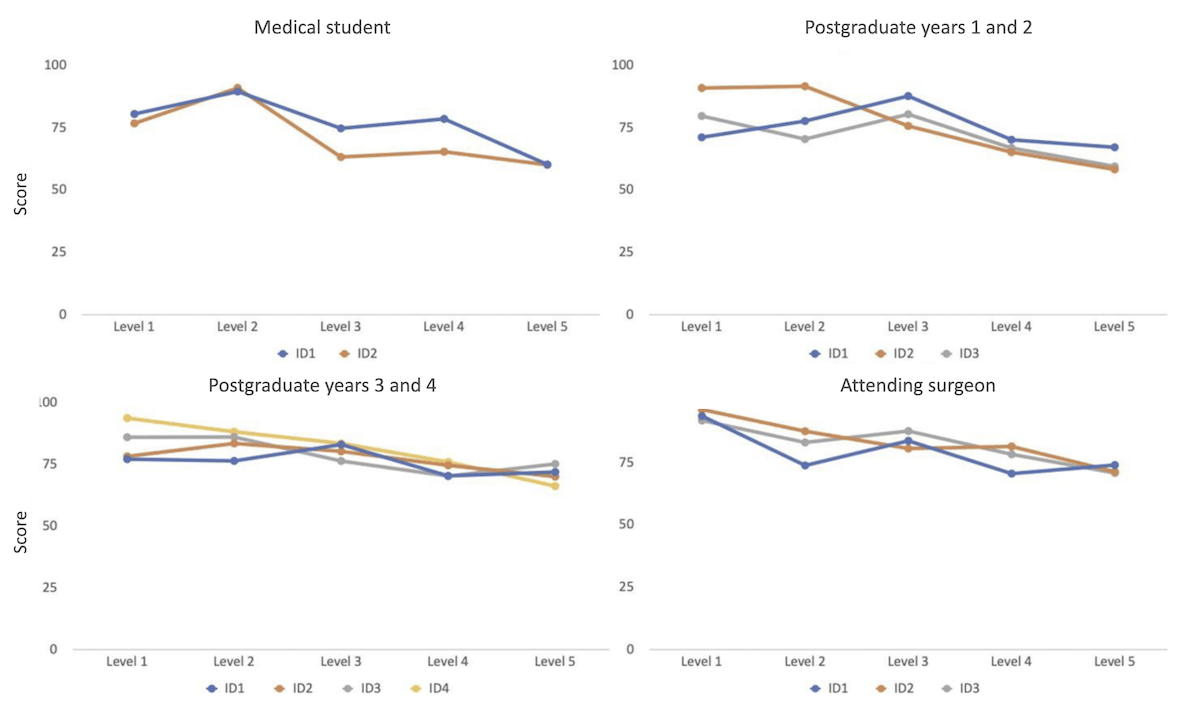
Average scores across academic expertise (medical student, postgraduate years 1 and 2, postgraduate years 3 and 4, and attending surgeon). Patterns and similarities can be seen in gameplay performance within each expertise group, highlighting the progression and consistency of scores.

In looking at individual players of a specific academic level, we see a relationship such that users with levels of experience exhibit comparable performance in passing levels and achieving scores. Specifically, no statistically significant difference was observed in the overall performance of participants within each group: medical students (*P*=.35), PGY1 and 2 residents (*P*=.43), PGY3 and 4 residents (*P*=.95), and fellows or attendings (*P*=.37; Figure 9). This finding suggests that LapBot can effectively measure skill levels appropriate to each stage of training, and any significant or predefined deviations from the expected performance could be used in a competency-based curriculum to tailor instruction and assessment.

These performance results validate the design of the LapBot app, showcasing its alignment with academic performance standards.

### Usability Feedback Survey

Beta testers completed a short feedback survey after completing the game. We describe the key takeaways below. In terms of the nonannotated surgical videos players could watch prior to choosing a target, players appreciated the use of videos as it helped them to contextualize the information. In terms of the AI feedback, users appreciated the feedback provided, particularly in the form of videos and the AR overlay of the AI, which helped guide their learning.

The design of the app and the interface were described as easy to use, intuitive, and visually appealing, and many users found the app to be addictive and enjoyable, with a fun interface and increasing difficulty level. Furthermore, the addition of a leaderboard was well-received, with users enjoying the competitive aspect of the app.

Additionally, the external survey data (n=13) produced results consistent with the in-app survey. Questions were asked on a Likert scale from 1 to 5 (with 1=low and 5=high). As shown in Table 1, most respondents found LapBot to be a fun way to learn LC (mean 4.77, SD 0.44) respondents also agreed that the app is easy to use (mean 4.62, SD 0.65) and navigate (mean 4.62, SD 0.65). Furthermore, respondents agreed that the feedback provided in the app was helpful for their learning process (mean 4.0, SD 1.0), and gamification elements motivated them to play more (mean 4.38, SD 0.87).

**Table 1:** External survey results on the LapBot game.

Question	Response (Likert scale), mean (SD)
The LapBot game was easy to use?	4.62 (0.65)
The LapBot game was easy to navigate?	4.62 (0.65)
LapBot is a fun way to learn about safe cholecystectomy?	4.77 (0.44)
How helpful is the answer key feedback provided by the game?	4.00 (1.00)
Gamification elements encourage playing?	4.38 (0.87)

## Discussion

### Principal Findings

In the beta testing of LapBot, 29 participants played the game, of which, 14 (48%) completed all 5 levels. This rate of abandonment is in line with those seen in mobile health apps (53%) [25], mobile apps in general (75%) [26], and serious games, which also have a high level of abandonment [27,28]. The cohort included medical students, trainees (PGY1-5), and attending surgeons. Flow theory models suggest that users experience optimal engagement in a game or “flow,” when the challenge of the game aligns with their skill level, leading to heightened focus and performance [29]. Maintaining this balance between challenge and skill level is important to ensure a state of flow, preventing frustration from overly difficult challenges and boredom from lack of stimulation [29]. Thus, game levels were intentionally designed to escalate in difficulty, a structure validated by participants’ increasing attempts as they progressed through the levels.

Player confidence, performance, and comparative analysis across different expertise levels revealed a positive correlation between academic proficiency and success, beginning to suggest LapBot’s alignment with educational standards. The in-app survey, involving 12 users, indicated high satisfaction with LapBot’s fun and effective learning experience, although some suggestions for feedback improvement were noted. Usability feedback and external survey data further supported the positive reception, with users appreciating the intuitive interface, addictive gameplay, and motivating game elements like leaderboards. Overall, the study demonstrates LapBot’s potential as a valuable educational resource, acknowledging both its strengths and areas for improvement based on user feedback.

### Limitations

The beta testing phase of LapBot presented in this paper primarily focused on assessing the gamification elements and design aspects for motivating and engaging users in learning about safe cholecystectomy. The current evaluation provides valuable insights into user experience, satisfaction, and the effectiveness of the game’s motivational features. However, the assessment is preliminary, as it primarily gauges user perceptions and participation without yet delving into a controlled examination of actual learning outcomes. To comprehensively understand the game’s impact, a more structured and controlled study is necessary, one that specifically measures the acquisition of knowledge and skills related to safe cholecystectomy. This future testing will be done to examine how the game contributes to learning objectives and, more critically, how that acquired knowledge translates into improved surgical decision-making and performance in real-world scenarios.

### Implications

The preliminary results from the gameplay scoring data suggest that LapBot could be useful in evaluating students’ progress and identifying individuals who may require additional lessons and practice before advancing to the next academic year. This will be an important aspect to study in a larger cohort, particularly with respect to the literature on competency-based curricula and proficiency assessment [30]. Surgical education is increasingly using competency-based curricula and evaluations, which prioritize skill and knowledge demonstration over time-based training. Nonetheless, a key challenge in implementing this approach is the uncertainty surrounding how to effectively monitor and evaluate skill acquisition and competency, facilitating interventions for those in need and certifying those who meet standards [31,32]. Our research is a step in this direction, as LapBot may provide a platform for objectively measuring trainees’ competencies through game scores and progress tracking. Furthermore, the results of this testing showed the validity of the designed gameplay elements, interface choices, and AI feedback. As part of our future work, further testing of the game with a larger user population will enable us to refine and establish more precise patterns based on users’ experience. This iterative process will allow us to develop robust and reliable measures that effectively distinguish players based on their achieving scores, thereby facilitating the implementation of a competency-based curriculum.

One of the contributions of this research lies in the integration of AI-generated feedback into a surgical training game. This strategy is novel in comparison to conventional methods that predominantly emphasize VR and serious gaming without significant integration of AI [33,34]. The use of AI-generated annotations as real-time feedback within the LapBot game aims to enhance learning. This integration helps trainees to practice in a dynamic, risk-free environment that mirrors real surgical scenarios, offering expert insights that contribute to safer and more proficient surgical practices. By integrating AI-generated feedback into surgical training, this research bridges the gap between AI capabilities and expert surgical knowledge. The AI models’ ability to identify safe and unsafe zones draws from a large data set of expert annotations. The game serves as a bridge that translates AI insights into actionable guidance for trainees, promoting safer and more effective surgical techniques.

### Conclusions

In the surgical domain, adverse events often occur due to errors in visual perception and judgments that lead to misinterpretation of anatomy [1]. The integration of gamification elements within the LapBot Safe Chole app presents an advancement in LC surgical education. By incorporating features such as scoring, leaderboards, animations, an intuitive interface, and effortless navigation, we aimed to provide users with an immersive and enjoyable learning experience. The app’s user-friendly design, combined with real-world surgical scenarios, encourages active decision-making, while the integration of AI annotations as feedback enhances the learning process. As users progress through various levels of the game, they receive scores and can compete against other players. This scoring system, coupled with competitive leaderboards, encourages players to improve their performance and increases engagement. In essence, the integration of game elements in LapBot aims to provide an exciting and interactive experiential learning environment.

Studying the use of serious games in LC offers a wealth of benefits that have the potential to mitigate some of the issues of the “see one, do one, teach one” surgical learning paradigm. Serious games provide an innovative platform for surgeons-in-training to practice complex procedures in a risk-free virtual environment, allowing them to hone their skills, improve hand-eye coordination, and enhance decision-making abilities. By simulating realistic scenarios and complications, serious games facilitate a dynamic learning experience that bridges the gap between theory and practice. This approach not only reduces the learning curve for novice surgeons but also offers experienced surgeons an avenue to refine their techniques and adapt to new advancements. Moreover, integrating serious games into LC training can potentially reduce the occurrence of errors during real-life surgeries, leading to safer procedures and better patient care overall.

The findings of our beta testing of the first prototype of LapBot Safe Chole underscore the effectiveness of LapBot as an engaging and efficient tool for exposing users to a wide range of surgical cases and facilitating skill development. By providing a fun game for users to engage with surgical scenarios, LapBot offers a valuable opportunity for users to enhance their surgical knowledge, gain practical insights, and improve their skills over time.

## Abbreviations

AI: artificial intelligence
AR: augmented reality
LC: laparoscopic cholecystectomy
PGY: postgraduate year
VR: virtual reality

## Data Availability

Data sharing is not applicable to this paper as no data sets were generated or analyzed during this study.
